# Increased One-Year Recurrent Ischemic Stroke after First-Ever Ischemic Stroke in Males with Benign Prostatic Hyperplasia

**DOI:** 10.3390/ijerph17155360

**Published:** 2020-07-25

**Authors:** Chun-Gu Cheng, Hsin Chu, Jiunn-Tay Lee, Wu-Chien Chien, Chun-An Cheng

**Affiliations:** 1 Department of Emergency, Taoyuan Armed Forces General Hospital, Taoyuan 32549, National Defense Medical Center, Taipei 11490, Taiwan; doc50015@yahoo.com.tw; 2 Department of Emergency Medicine, Tri-Service General Hospital, National Defense Medical Center, Taipei 11490, Taiwan; 3 Department of Emergency and Critical Medicine, Wan Fang Hospital, Taipei Medical University, Taipei 11696, Taiwan; 4 Department of Neurology, Tri-Service General Hospital, National Defense Medical Center, Taipei 11490, Taiwan; hrchu@ndmctsgh.edu.tw (H.C.); jiunntay@gmail.com (J.-T.L.); 5 Institute of Aerospace and Undersea Medicine, National Defense Medical Center, Taipei 11490, Taiwan; 6 Department of Medical Research, Tri-Service General Hospital, National Defense Medical Center, Taipei 11490, Taiwan; chienwu@ndmctsgh.edu.tw; 7 School of Public Health, National Defense Medical Center, Taipei 11490, Taiwan; 8 Graduate Institute of Life Sciences, National Defense Medical Center, Taipei 11490, Taiwan

**Keywords:** benign prostatic hyperplasia, one-year recurrent ischemic stroke, cerebral autoregulation dysfunction

## Abstract

(1) Background: Patients with benign prostatic hyperplasia (BPH) were questioned about quality of life and sleep. Most BPH patients were treated with alpha-1 adrenergic receptor antagonists, which could improve cerebral blood flow for 1–2 months. Patients with ischemic stroke (IS) could experience cerebral autoregulation impairment for six months. The relationship between BPH and recurrent IS remains unclear. The aim of this study was to determine the risk of one-year recurrent IS conferred by BPH. (2) Methods: We used data from the Taiwanese National Health Insurance Database to identify newly diagnosed IS cases entered from 1 January 2008 to 31 December 2008. Patients were followed until the recurrent IS event or 365 days after the first hospitalization. The risk factors associated with one-year recurrent IS were assessed using Cox proportional hazards regression. (3) Results: Patients with BPH had a higher risk of recurrent IS (12.11% versus 8.15%) (adjusted hazard ratio (HR): 1.352; 95% confidence interval (CI): 1.028–1.78, *p* = 0.031). Other risk factors included hyperlipidemia (adjusted HR: 1.338; 95% CI: 1.022–1.751, *p* = 0.034), coronary artery disease (adjusted HR: 1.487; 95% CI: 1.128–1.961, *p* = 0.005), chronic obstructive pulmonary disease (adjusted HR: 1.499; 95% CI: 1.075–2.091, *p* = 0.017), and chronic kidney disease (adjusted HR: 1.523; 95% CI: 1.033–2.244, *p* = 0.033). (4) Conclusion: Patients with BPH who had these risk factors had an increased risk of one-year recurrent IS. The modification of risk factors may prevent recurrent IS.

## 1. Introduction

Incidents of recurrent ischemic stroke (IS) are higher than that of hemorrhagic stroke in Asia [1]. Recurrent IS is related to older age, more atherosclerotic risk factors, and cigarette smoking or drinking habits [2]. Recurrent IS is a challenge in terms of treatment and extended care; risk factors need to be recognized, and aggressive strategies for prevention must be considered. The traditional risk factors for atherosclerosis are well known, but some undetectable risk factors must still be identified. The patients with IS have cerebral autoregulation impairment and parasympathetic dysfunction with sympathetic overactivity [3]. Mild chronic IS for more than six months is associated with decreased cerebral autoregulation [4].

Benign prostatic hyperplasia (BPH) is a noncancerous enlargement of the prostate gland that causes symptoms by obstructing the urethra. Male patients with BPH were questioned about their quality of life and whether their condition disturbed their sleep. The majority of patients with BPH were treated with alpha-1 adrenergic receptor antagonists. A previous study found that alpha-1 adrenergic receptor antagonist therapy could improve cerebral blood flow after treatment for 4–8 weeks [5].

A past study revealed that symptoms of BPH were correlated with traditional cardiovascular risk factors. Endothelial dysfunction, defined as decreased levels of flow-mediated vasodilation in the brachial artery, was associated with an increased risk of moderate-to-severe lower urinary tract symptoms (LUTS) in men. Endothelial dysfunction, which initially occurs in the pathogenesis of atherosclerosis, was associated with LUTS in men. Moderate-to-severe LUTS was associated with the prevalence of coronary heart disease in men but not in women [6]. Nocturia was the main symptom of BPH that increased sympathetic activity and decreased nondip blood pressure during the night [1]. The patients with severe LUTS had increased long-term risk of major adverse cardiovascular events compared with those who were symptom-free or had mild symptoms of BPH [7]. A pooling study found that LUTS in patients free of cardiovascular disease were not associated with cardiovascular events in elderly patients [8]. The relationship between BPH and recurrent IS is unknown, and our aim is to determine if recurrent IS will be increased within one-year post-ischemic stroke in patients with BPH. We used the Taiwanese National Health Insurance Database to survey patients with recurrent IS within one year after first-ever IS.

We hypothesized that male IS patients with BPH had an increased risk of one-year recurrent IS. Patients with BPH had a 35% increased risk of recurrent IS in our study. When males with BPH suffer first-ever IS, physicians and patients must focus on the BPH symptoms and initiate aggressive lifestyle modification and therapy for atherosclerotic risk factors to reduce the occurrence of recurrent IS.

## 2. Materials and Methods 

### 2.1. Database

The Taiwanese National Health Insurance is a single-payer health care system funded by the government and includes approximately 99% of the nation’s 23 million residents. The Longitudinal National Health Insurance Research Database (LNHID) released a cohort dataset consisting of two million randomly sampled people. In this cohort dataset, each patient’s original identification number has been encrypted to protect privacy. The National Health Insurance Research Database (NHIRD) contains outpatient and inpatient medical payment records. There are up to three International Classification of Disease, Ninth Revision, Clinical Modification (ICD-9-CM) outpatient diagnosis codes and five ICD-9-CM inpatient diagnosis codes. This study used the LNHID from 2008 to 2009 and observed the occurrence of recurrent IS within one year in ischemic stroke patients in 2008. This study was approved by the Ethics Institutional Review Board of the Tri-Service General Hospital (TSGHIRB 2-103-05-050).

### 2.2. Design

**Figure 1:**
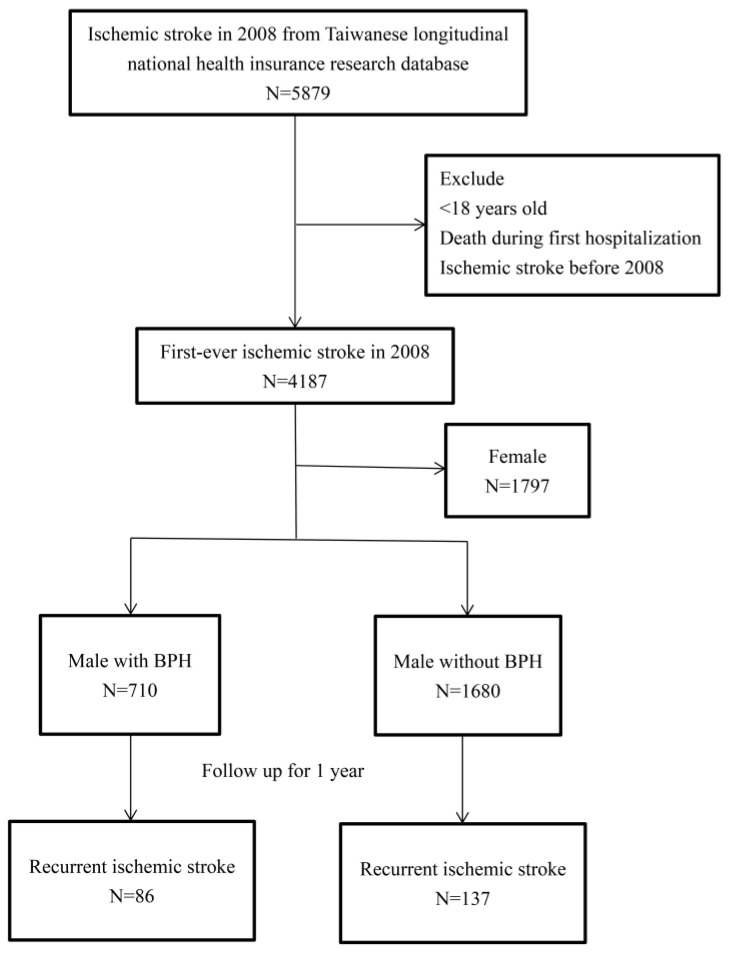
The flowchart of this study. BPH: benign prostatic hyperplasia.

We used outpatient and inpatient data from the LNHID to identify newly diagnosed IS cases that were entered from 1 January 2008 to 31 December 2008 in Taiwan. The database contained patient identification numbers, outpatient visits, and admission dates. We retrieved data for patients with a first diagnosis of IS (ICD-9-CM diagnostic code 433–437). The events of recurrent IS were defined (ICD-9-CM diagnostic codes 433–437) after discharge from the first admission. Patients were followed until the recurrent IS event or 365 days after the first admission. We excluded patients who were younger than 18 years of age, died during the first hospitalization for IS, female, or were diagnosed before 2008. The date of the first diagnosis of IS was defined as the index date. The flow chart of this study is shown in Figure 1.

Comorbidities mapped by ICD-9-CM codes included hypertension (401–405), diabetes mellitus (250), atrial fibrillation (427.31), hyperlipidemia (272), coronary artery disease (410–414), congestive heart failure (428), chronic kidney disease (580–589), peripheral artery obstructive disease (443), chronic obstructive pulmonary disease (491, 492, 496), BPH (600), and hypotension (458).

### 2.3. Statistical Analyses

Continuous variables were compared using the Student’s *t*-test and are displayed as the mean ± standard deviation. Categorical variables were compared using the chi-square test and are displayed as percentages. We performed a collinear analysis and showed 0.88 for hypertension with hyperlipidemia, 0.9 for coronary artery disease with atrial fibrillation, and 0.84 for hypotension with BPH. The risk factors associated with one-year recurrent IS or mortality were assessed using a Cox proportional hazard regression with forward stepwise selection to reduce collinearity. The statistical significance was set at *p* < 0.05. The statistical analysis was performed using SPSS version 21 software (Asia Analytics Taiwan Ltd., Taipei, Taiwan).

## 3. Results

**Figure 2:**
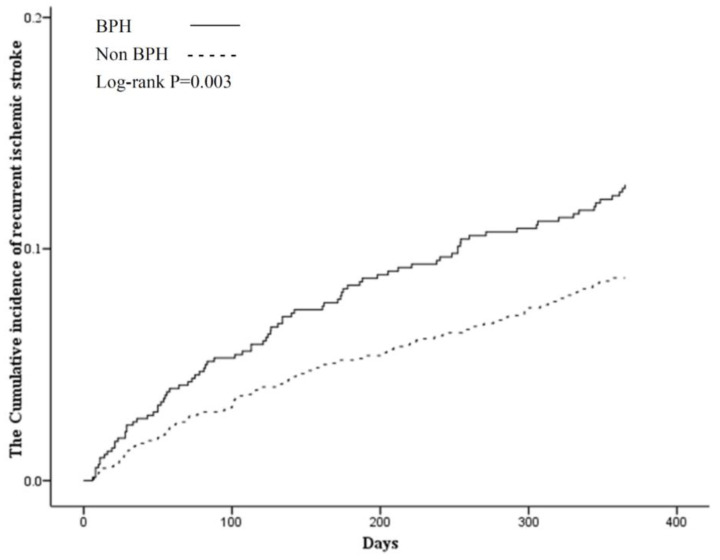
Kaplan–Meier curve for cumulative incidence of one-year recurrent ischemic stroke stratified by benign prostatic hyperplasia using the log-rank test. BPH: benign prostatic hyperplasia.

The incidence of one-year recurrent IS was 9.22% (223/2390) after the first-ever IS. Patients who experienced recurrent IS within one year of the first-ever IS more frequently had BPH, hyperlipidemia, coronary artery disease, chronic obstructive pulmonary disease, chronic kidney disease, and hypotension (Table 1). The cumulative incidence of one-year recurrent IS was higher in BPH group (log-rank test: *p* = 0.003) (Figure 2).

The risk factors associated with one-year recurrent IS, assessed using multivariate Cox regression with forward stepwise selection, were BPH (adjusted hazard ratio (HR): 1.352; 95% confidence interval (CI): 1.028–1.78, *p* = 0.031), hyperlipidemia (adjusted HR: 1.338; 95% CI: 1.022–1.751, *p* = 0.034), coronary artery disease (adjusted HR: 1.487; 95% CI: 1.128–1.961, *p* = 0.005), chronic obstructive pulmonary disease (adjusted HR:1.499; 95% CI: 1.075–2.091, *p* = 0.017), and chronic kidney disease (adjusted HR: 1.523; 95% CI: 1.033–2.244, *p* = 0.033) (Table 2). Our study found that age was an independent risk factor for BPH with an adjusted OR of 1.061 (95% CI: 1.052–1.07, *p* < 0.001). Other factors were hypertension (adjusted OR 1.421; 95% CI: 1.135–1.804, *p* = 0.002) and coronary artery disease (adjusted OR 1.333; 95% CI: 1.093–1.625, *p* = 0.005).

There were 309 (12.93%) male patients who died within one year after first-ever IS. The risk factors for one-year mortality were age (adjusted HR: 1.052; 95% CI: 1.04–1.064, *p* < 0.001), diabetes mellitus (adjusted HR: 1.678; 95% CI: 1.326–2.124, *p* < 0.001), chronic obstructive pulmonary disease (adjusted HR: 1.343; 95% CI: 1.03–1.751, *p* = 0.03), and chronic kidney disease (adjusted HR: 2.547; 95% CI: 1.935–3.352, *p* < 0.001) (Table 3).

## 4. Discussion

We found that BPH was associated with recurrent IS within one-year post-IS. First-ever IS with multiple comorbid conditions could cause recurrent IS within one year. Some patients who recovered from IS after treatment and still experience poor health conditions need to modify their health behaviors to prevent recurrent IS.

The circadian rhythm of BP is negatively affected in BPH patients because of increased nocturia episodes with sleep disturbances and sympathetic overactivity; nondipping blood pressure increases the risk of cardiovascular disease morbidity and mortality [9]. Severe LUTS with BPH increases the risk of major adverse cardiovascular events with an odds ratio of 1.68 [7]. A previous study found that alpha-1 adrenergic receptor antagonist therapy in elderly patients with BPH caused initial hypotension with increased IS within three weeks but improved cerebral blood flow without influencing IS later [2]. Increased atherosclerotic burden and endothelial dysfunction were observed in BPH patients in a recent study [6]. Furthermore, patients with IS had cerebral autoregulation impairment that persisted for six months in a previous study [10]. We found that BPH was associated with advanced age; hypertension and coronary artery disease shared risk factors of atherosclerosis. The older IS patients carried higher risk of mortality in recurrent IS. The previous studies found doxaben treatment for chronic IS patients with hypertension increased cerebral blood flow that reduced recurrent IS [5]. A past study of hypertension treated with doxaben in Chinese patients compared to diuretic treatment decreased lipid levels [11] and normalized platelet function [12]. Aggressive antiplatelet and lipid-lowering agent prescriptions for IS patients that can reduce coronary artery disease correlated to recurrent IS. BPH seems to have been an unrecognized risk factor of recurrent IS in the past. 

We surveyed first-ever IS patients to find a relationship between BPH and one-year recurrent IS. Our study found that patients with BPH exhibited a crude odds ratio (OR) of 2.347 (95% CI: 1.409–3.907, *p* = 0.001) and experienced more hypotension (4.23% versus 1.88%, *p* = 0.001). The hypotension carried a two-fold risk of recurrent IS in univariate analysis. There were 571 patients who used alpha blockers, 31 patients who used 5-alpha reductase inhibitors, and 137 patients without medication records due to random selection of medications selection. There were 542 patients with BPH treated by single-type medication with alpha blockers that carried a higher risk of recurrent IS of crude HR 1.521 (95% CI: 1.135–2.037, *p* = 0.005), a higher risk of hypotension of crude OR 1.932 (95% CI: 1.083–3.45, *p* = 0.026), and 29 patients with double-type medications (alpha blockers and 5-alpha reductase inhibitors) with an insignificant difference (crude HR 1.672; 95% CI: 0.619–4.519, *p* = 0.311) not related to hypotension (crude OR 1.9; 95% CI: 0.25–14.408, *p* = 0.535). In a previous study of chronic IS patients with hypertension treated with doxaben, blood pressure of 150 mmHg reduced to 130 mmHg without hypotension events, and patients showed increased cerebral blood flow [5]. The past study found parasympathetic impairment in the acute and chronic phases of IS [13]. The central cholinergic effect increased cerebral blood flow for orthostatic stress-induced hypotension [14]. The potential mechanism was the alpha blocker treatment for BPH may induce hypotension; parasympathetic cholinergic dysfunction in IS patients induced cerebral hypoperfusion. The potential reasons for increased recurrent IS are a lack of improvement in cerebral perfusion in IS and a tendency towards hypotension.

The survival and recurrence rates in IS patients were higher than those in hemorrhagic stroke patients in a Singapore study [15]. The prognosis for survival of IS was important to patients, their families, and clinicians. Through aggressive preventive strategies and lifestyle modification, recurrent IS trends were reduced from 9.6% to 7.8% from 2000 to 2011 in Taiwan [16]. Recurrent IS was shown to be related to advanced age, hypertension, diabetes mellitus, hyperlipidemia, obstructive sleep apnea, smoking, and alcohol use [17]. Although these risk factors are important in recurrent IS pathophysiology and clinical presentation, no single theory is sufficient to provide an adequate explanation for all the properties of recurrent IS. The Stroke Prevention by Aggressive Reduction in Cholesterol Levels (SPARCL) trial provides evidence for the role of statins in reducing risk for future cardiovascular events in all patients with stroke, independent of cholesterol levels, and even at low-density lipoprotein cholesterol levels ≤100 mg/dL [18]. Our study showed similar results. In the Framingham study, the age-adjusted two-year incidence of stroke was more than double in the presence of coronary artery disease [19]. Our study found that coronary artery disease carried a 1.5-fold risk in one-year recurrent IS, which was lower than that of the past study. The potential reason was that BPH carried risk when interacting with other risk factors and adequate antiplatelet therapy. The main pathophysiological mechanisms of chronic obstructive pulmonary disease are systemic inflammation, hypoxia, hypercapnia, and oxidative stress [20]. Patients with chronic obstructive pulmonary disease tended to have incident atrial fibrillation [21], which could easily induce embolic stroke. A meta-analysis showed that chronic obstructive pulmonary disease caused IS with an HR of 1.31 (1.03–1.66) [20]. Our study showed a similar result of a 1.5-fold risk of one-year recurrent IS. One-year noncardioembolic stroke was related to old age, lower high-density lipoproteins, and chronic kidney disease with an HR 1.73 (95% CI: 1.03–2.90) in Japan [22]. Higher mortality risk was observed in patients with chronic kidney disease. Our study showed a lower risk of chronic kidney disease in one-year recurrent IS risk with the competing risk of death.

Age is a nonmodifiable risk factor for recurrent IS [15]. Prostate volume increases with age, suggesting a prostate growth rate of 2.0–2.5% per year in older men [23]. One study found increasing age-induced atherosclerosis and prostatic enlargement [24]. A one-year recurrent IS study in Sweden found that patients over 75 years of age had an increased risk [25]. However, our study did not show that age was related to one-year recurrent IS; a potential reason may be that older IS patients had higher mortality with an HR of 1.052 (95% CI: 1.04–1.064) competing against the risk. Diabetes may induce vascular injury through nitric oxide-dependent vasodilation, and hyperglycemia can cause ischemia in the brain by anaerobic metabolism with lactate accumulation and intracellular acidosis [26]. A Swedish study found that diabetes mellitus can increase one-year recurrent IS risk with an HR 1.18 (1.12–1.25) [25]. Our study showed no association of recurrent IS with diabetes, which was associated with a 1.7-fold increase in mortality risk. The trials of statins and blood pressure-lowering therapy suggest a new paradigm for secondary stroke prevention. The Perindopril Protection Against Recurrent Stroke Study provides active blood pressure-lowering therapy with a 28% relative risk reduction in recurrent stroke [27]. Our study showed no significant risk of hypertension. The potential reason may be adequate treatment according to the diagnostic codes in the claims data and alpha blockers with an antihypertensive effect. Major hemispheric stroke syndrome, atherothrombotic stroke mechanism, and atrial fibrillation were independent predictors of early recurrence [28]. Anticoagulant therapy reduced the risk of recurrent stroke within six months of the first-ever IS in Taiwanese patients with an odds ratio of 0.55 (95% CI: 0.44–0.8) [29] and within one year of the first-ever IS in Swedish patients with an HR of 0.80 (95% CI: 0.73–0.88) [25]. Our study did not show a significant increase in the risk of atrial fibrillation. Congestive heart failure is the end stage of cardiac disease with poor prognosis. The risk of IS in congestive heart failure patients was related to the coexistence of vascular risk factors related to a higher atherosclerotic burden and endothelial dysfunction with embolism or cerebral hypoperfusion. A previous study found that congestive heart failure was independently associated with short-term stroke recurrences at one week (adjusted OR: 2.66) and at three months (2.41) [30]. However, our study showed no association with congestive heart failure, and the potential reason may be related to the low prevalence of approximately 6% in our study.

It is important to recognize the mechanisms involved in recurrent IS. For secondary prevention of recurrent IS, optimal control of all vascular risk factors is essential. Although antiplatelet agents, anticoagulant agents, and increased statin use were noted in previous surveys [16], they are not optimal for the control of risk factors. Mechanisms of treatment of conditions, such as carotid stenosis or atrial fibrillation, need to be identified in prevention of IS recurrence. Treatment of carotid stenosis with a carotid stent or carotid endarterectomy can reduce recurrent IS [31]. New oral anticoagulant agents, the costs of which have been covered by insurance since 2012, reduced recurrent IS in atrial fibrillation patients [32]. Statin drugs have been used to treat hyperlipidemia, and they not only improved endothelial dysfunction but also reduced the risk of cardiovascular events [33]. Correction of hypoxia and acid–base imbalance is an effective therapy for chronic obstructive pulmonary disease patients. Cardioselective β-blockers can be prescribed for chronic obstructive pulmonary disease with atrial fibrillation [34]. Moderate-to-vigorous physical activity, compared with a sedentary lifestyle, reduced the risk of BPH by 25% [35]. Recurrent IS patients after their first-ever minor noncardioembolic IS engaged in lower levels of moderate-to-vigorous physical activity and had higher levels of visceral fat than recurrent IS-free patients in Japan [36]. Encouraging more aggressive physical activity to reduce obesity could prevent recurrent IS as higher levels of activity provide an increasingly protective effect. More aggressive lifestyle modification may prevent recurrent IS. 

The patients with BPH used alpha-1 blockers to improve the LUTS symptoms that will enhance cerebral flow through cerebral autoregulation in hypertension patients for weeks [5]. The collinear analysis showed 0.84 for hypotension with BPH, but there was a lower prevalence of hypotension that was not related to recurrent IS after being adjusted for other factors. The previous study found a higher risk (HR 1.71, 95% CI: 1.10–1.30) of hypotension with the use of alpha blockers in older women [37]. For patients with IS and impaired parasympathetic cerebral autoregulation, alpha-1 blocker treatment for BPH carried no benefit but rather harm. If IS patients with BPH were given treatment with 5-alpha reductase inhibitors rather than alpha-1 blockers, they could avoid potential hypotension to reduce recurrent IS [38].

There were some limitations in our study. First, body mass index, smoking, and alcohol use were not available in the payment claims dataset of NHIRD. These factors may have an impact on recurrent IS risk. Second, a previous study found previous IS or transient ischemic attack, large vessel disease, cortical lesion, and multiple infarctions to be predictive of recurrent IS [39]. However, our study did not review radiological information in the claims data, which could influence the prediction of recurrent IS, unlike IS subtypes. Third, this study found BPH to be related to recurrent IS in the Taiwanese Chinese population. Whether this finding is also supported in other populations should be corroborated in the future. The higher prevalence of intracranial atherosclerosis was noted in Asia; it may be related to BPH-induced recurrent IS in the Chinese population. There were 46.6% intracranial atherosclerosis instances and 9.11% extracranial carotid stenosis instances in Chinese IS patients; the severity of intracranial artery disease is related to one-year recurrent IS [40]. Further clinical study is needed to confirm the relationship between BPH with hypotension and intracranial atherosclerosis in recurrent IS.

## 5. Conclusions

This population observation study provides a retrospective investigation of the association of BPH with one-year recurrent IS after first-ever IS. The risk factors for recurrent IS include BPH, hyperlipidemia, coronary artery disease, chronic obstructive pulmonary disease, and chronic kidney disease. Physicians must recognize stronger risk factors and provide early and continuous specific preventive strategies to reduce recurrent IS after acute IS.

## Figures and Tables

**Table 1:** The characteristics between recurrent ischemic stroke and non-recurrent ischemic stroke.

Risk Factors	Recurrent Ischemic Stroke (223)	Recurrent Ischemic Stroke Free (2167)	*p*
Age	69.25 ± 11.22	67.85 ± 13.10	0.082
Benign prostatic hyperplasia	86 (38.57%)	624 (28.8%)	0.002 *
Hyperlipidemia	95 (42.60%)	765 (35.30%)	0.031 *
Chronic obstructive pulmonary disease	49 (21.97%)	320 (14.77%)	0.005 *
Chronic kidney disease	31 (13.90%)	193 (8.91%)	0.015 *
Atrial fibrillation	27 (12.11%)	230 (10.61%)	0.493
Hypertension	176 (78.92%)	1649 (76.10%)	0.344
Diabetes mellitus	100 (44.84%)	863 (39.82%)	0.146
Coronary artery disease	90 (40.36%)	614 (28.33%)	<0.001 *
Peripheral arterial occlusive disease	6 (2.69%)	41 (1.89%)	0.413
Congestive heart failure	14 (6.28%)	133 (6.13%)	0.934
Hypotension	11 (4.93%)	50 (2.31%)	0.025 *

* *p* < 0.05.

**Table 2:** Risk factors for one-year recurrent ischemic stroke.

Risk Factors	Crude Hazard Ratio (95% C.I.)	*p*	Adjusted Hazard Ratio (95% C.I.)	*p*
Age	1.011 (1.001–1.022)	0.033 *		
Benign prostatic hyperplasia	1.502 (1.147–1.967)	0.003 *	1.352 (1.028–1.78)	0.031 *
Hyperlipidemia	1.270 (0.974–1.655)	0.078 *	1.338 (1.022–1.751)	0.034 *
Chronic obstructive pulmonary disease	1.720 (1.253–2.362)	0.001 *	1.499 (1.075–2.091)	0.017 *
Chronic kidney disease	1.805 (1.235–2.638)	0.002 *	1.523 (1.033–2.244)	0.033 *
Atrial fibrillation	1.190 (0.796–1.780)	0.396		
Hypertension	1.119 (0.811–1.543)	0.494		
Diabetes mellitus	1.248 (0.958–1.625)	0.1		
Coronary artery disease	1.709 (1.308–2.233)	<0.001 *	1.487 (1.128–1.961)	0.005 *
Peripheral arterial occlusive disease	1.483 (0.659–3.338)	0.341		
Congestive heart failure	1.070 (0.623–1.839)	0.806		
Hypotension	1.987 (1.084–3.643)	0.044 *		

* *p* < 0.05.

**Table 3:** Risk factors for one-year mortality after adjusted with other risk factors.

Risk Factors	Adjusted Hazard Ratio (95% Confidence Interval)	*p*
Age	1.052 (1.04–1.064)	<0.001 *
Diabetes mellitus	1.678 (1.326–2.124)	<0.001 *
Chronic obstructive pulmonary disease	1.343 (1.03–1.751)	0.03 *
Chronic kidney disease	2.547 (1.935–3.352)	<0.001 *

* *p* < 0.05.
